# A Novel Radiographic Finding of Intracapsular Heterotopic Ossification: A Report of Two Cases

**DOI:** 10.7759/cureus.11372

**Published:** 2020-11-07

**Authors:** Holly J Wilson, Parker B Goodell, Robert C Kollmorgen

**Affiliations:** 1 Research, University of California San Francisco, Fresno, Fresno, USA; 2 Orthopaedic Surgery, University of California San Francisco, Fresno, Fresno, USA; 3 Hip Preservation and Sports Medicine, University of California San Francisco, Fresno, Fresno, USA

**Keywords:** revision hip arthroscopy, heterotopic ossification, capsular reconstruction

## Abstract

Symptomatic heterotopic ossification (HO) is a complication of hip arthroscopy that occurs in less than 1% of cases. To our knowledge, there are no reported cases of symptomatic intracapsular HO. We present 2 patients with a radiographic finding associated with intracapsular HO. Both patients underwent revision hip arthroscopy and required capsular reconstruction due to void of the iliofemoral ligament following excision of HO. We believe this radiographic finding may be useful to hip preservationists as it may be associated with capsular deficiency necessitating capsular reconstruction upon revision hip arthroscopy.

## Introduction

Heterotopic ossification (HO) is a common complication following hip arthroscopy with an incidence of up to 44% [1, 2]. Reported risk factors include male gender, femoral neck osteochondroplasty, and unrepaired capsulotomy [1-4]. Current treatments range from nonoperative management of asymptomatic lesions, ultrasound guided lavage for a small symptomatic HO lesion, and revision surgery for excision of painful lesions [5]. Currently, there are no reported cases of intracapsular HO that required capsular reconstruction upon excision.

Heterotopic ossification has been shown to form in areas where an inflammatory process is occurring [1, 4, 6]. This results in formation of bone in abnormal anatomic locations [1, 3, 6]. In hip arthroscopy, performing a femoral osteochondroplasty has been shown to increase the risk of HO formation postoperatively [1, 2, 4]. The literature has also shown that larger cam resections may be at higher risk of developing HO [1, 3, 4]. HO has most commonly been reported forming at the anterior and lateral aspects of the hip [1, 3]. Anterior HO tends to be more symptomatic as it can cause impingement. However, in general, symptomatic HO is a rare complication which occurs in less than 1% of cases [1].

Previously described techniques for the operative treatment of symptomatic HO include open resection and arthroscopic excision of the painful lesion [1-4, 7, 8]. Revision arthroscopy with HO excision has been shown to improve patient reported outcome measures (PROMs) with an average of a 20 point improvement at a mean of 1.5 years using the modified Harris hip score [1, 4]. We present, to our knowledge, the first reported cases of symptomatic intracapsular HO that required arthroscopic excision in conjunction with capsular reconstruction due to capsular insufficiency. This radiographic finding may indicate the need for capsular reconstruction.

## Case presentation

Case 1 

**Figure 1:**
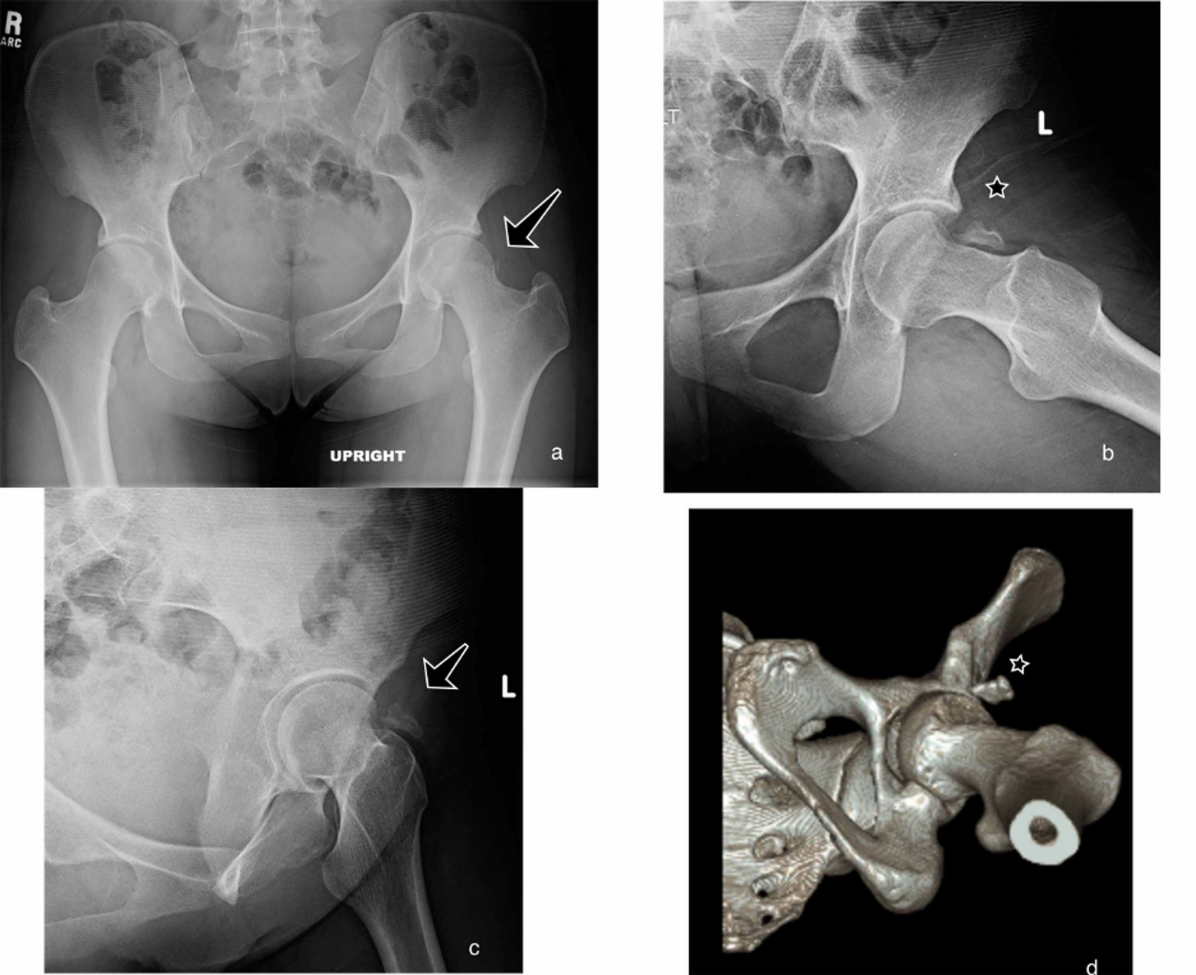
Case 1: Preoperative Imaging (a) AP pelvis with HO seen on the left hip (arrow). (b) Modified Dunn view showing grade 1 HO in the hip capsule (star). (c) False-profile view showing HO (arrow). (d) 3D reconstruction CT-image of the left hip demonstrating HO (star).

A 21-year-old female, with a history of 3 prior hip arthroscopies by outside surgeons, presented with persistent left hip pain. The patient had previously tried therapy and injections without resolution of pain. Her exam showed a synovitic hip with decreased range of motion (ROM), positive impingement tests, and evidence of instability with a decreased spring back sign and increased external rotation at 90 degrees of hip flexion. Preoperative imaging included plain radiographs and a three-dimensional (3D) computed tomography (CT) scan showing an over-resected femoral head and HO (Figure 1). The HO was classified as Brooker Grade 1 using the Brooker Classification [9]. Radiographic findings are shown in Table 1. After discussion of treatment options, the patient elected to proceed with revision hip arthroscopy. 

**Table 1:** Pre- and Postoperative Radiographic Findings Lateral Center Edge Angle (LCEA), Anterior Center Edge Angle (ACEA), Over Resection (OR), Residual Structural Disease (RSD), Heterotopic Ossification (HO), Anterior Inferior Iliac Spine (AIIS), Femoral Neck Version (FVN).

	Patient 1		Patient 2	
Radiographic Findings	Pre-Op	Post-Op	Pre-Op	Post-Op
LCEA	37	30	25	25
ACEA	34	29	28	27
Tonnis Angle	-2	4	11	11
Tonnis Grade	1	1	1	1
Alpha Angle	52	35	74	59
OR of femoral head	Yes	Yes	Yes	Yes
RSD present	Yes	No	Yes	No
HO present	Yes	No	Yes	No
AIIS type	1		2	
Acetabular Version				
1 o'clock	6		12	
2 o'clock	16		20	
3 o'clock	20		29	
FNV	9		22	

Patient Reported Outcome Measurement Information Systems Computer Adaptive Testing (PROMIS CAT) measures of: physical function (PF), anxiety (AX), depression (DP), pain interference (PI), ability to participate in social roles (SR), global health (GH), and pain intensity (PIY) were recorded preoperatively. PROMIS questions are formatted on a 1-5 point Likert scale, and a raw score is obtained. The raw score, total value based on the patient answers, is then converted to a standardized score based on the U.S. general population to generate the T-score. The mean standardized T-score for all measures is 50 with a standard deviation (SD) of 10. Significant function or dysfunction is determined by the question asked and a score above or below one SD [10, 11]. 

**Figure 2:**
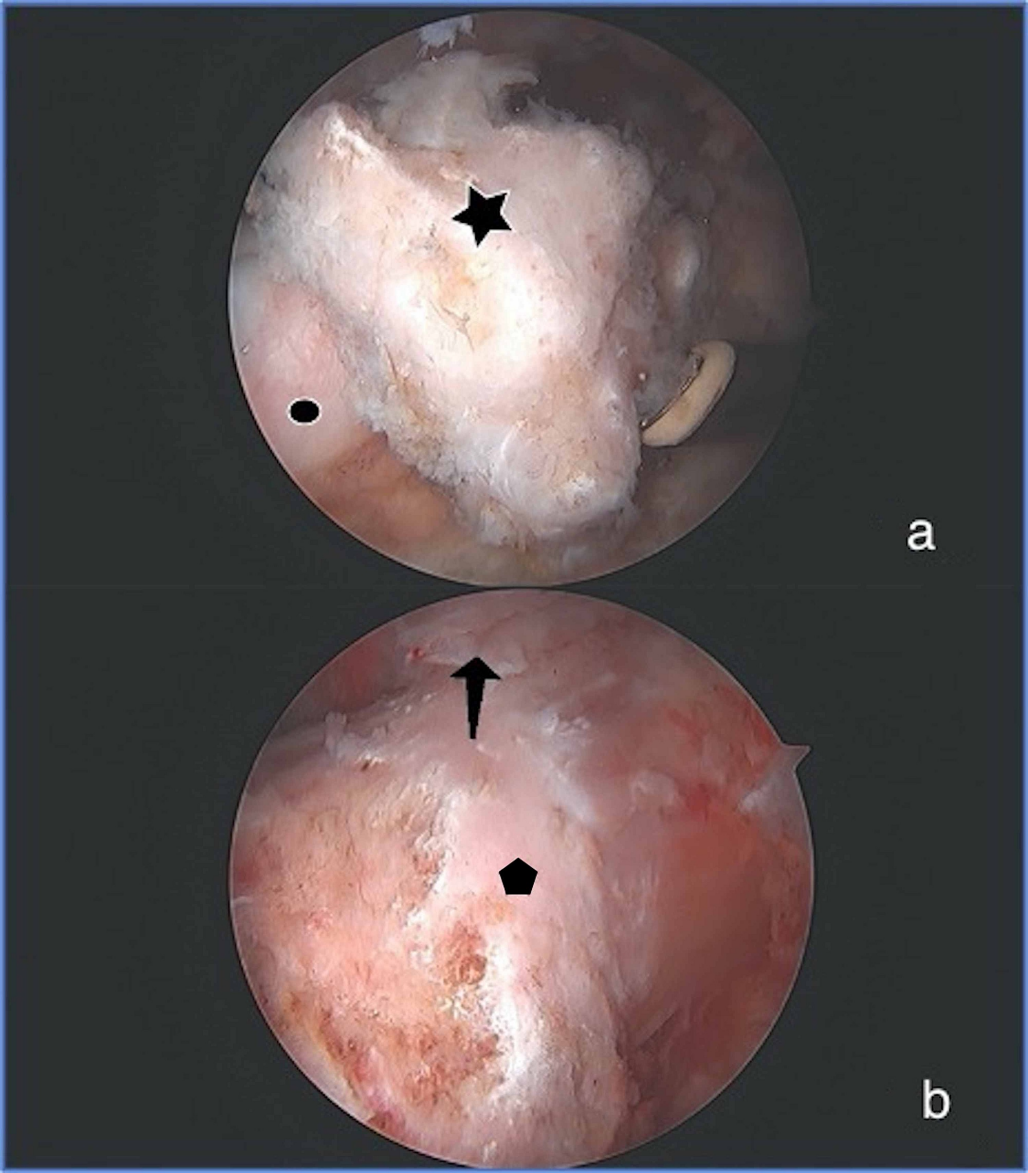
Arthroscopic Findings From Case 1 All images are of a left hip viewed from the anterolateral portal. (a) Heterotopic bone (star) of patient 1 which is intrasubstance within the capsule over the femoral neck (circle). (b) After HO resection in patient 1 an incompetent iliofemoral ligament was noted. The arrow indicates the remnant iliofemoral ligament distally. Residual disease on the anterolateral femoral neck was found (hexagon).

**Figure 3:**
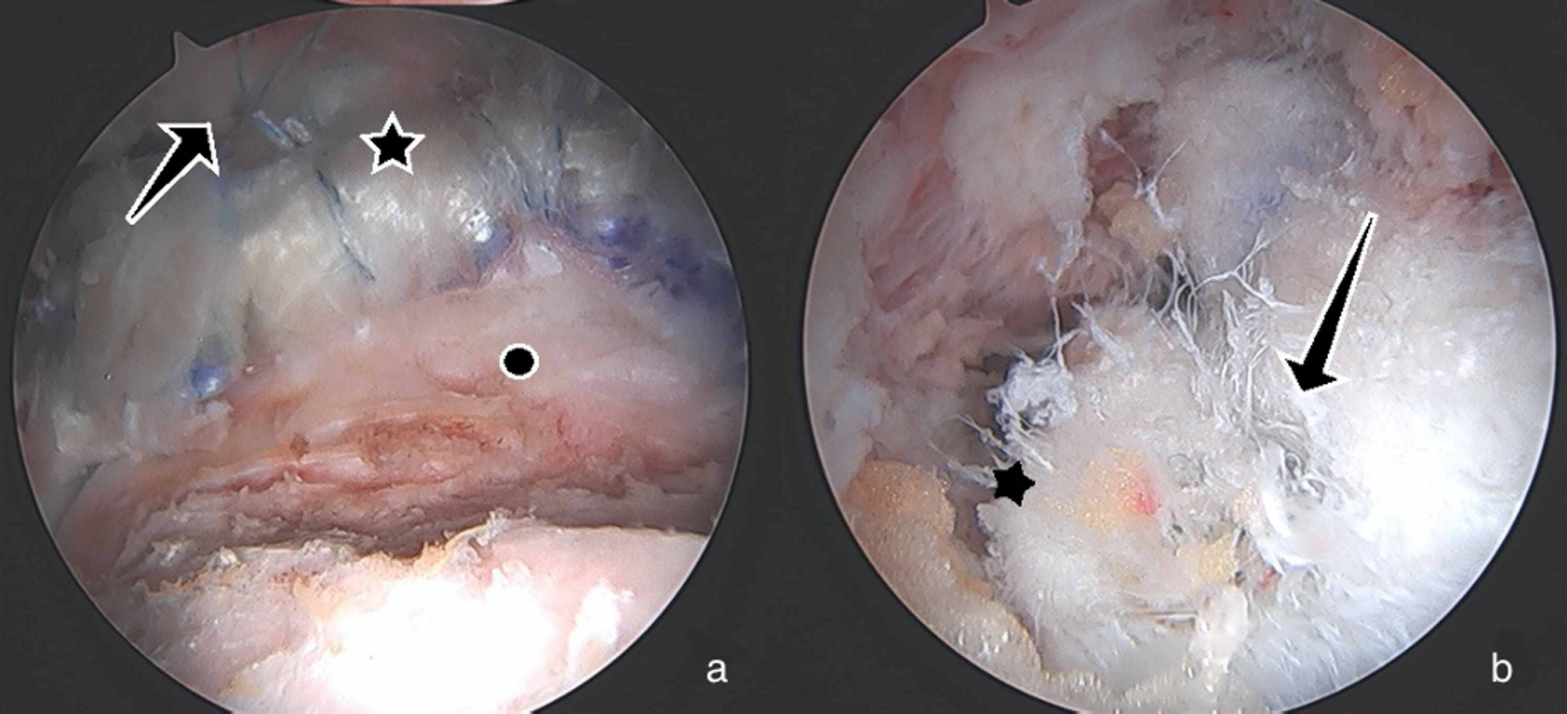
Arthroscopic Images from Case 1 Images are of a left hip. (a) Completed labral allograft reconstruction (star), viewed from the midanterolateral portal, with acetabular anchors for capsular reconstruction (arrow); the circle shows the over-resection of the femoral head from prior surgery. (b) Viewed from the anterolateral portal. The star designates the proximal medial portion of the capsular reconstruction; the arrow shows the dermal allograft.

**Figure 4:**
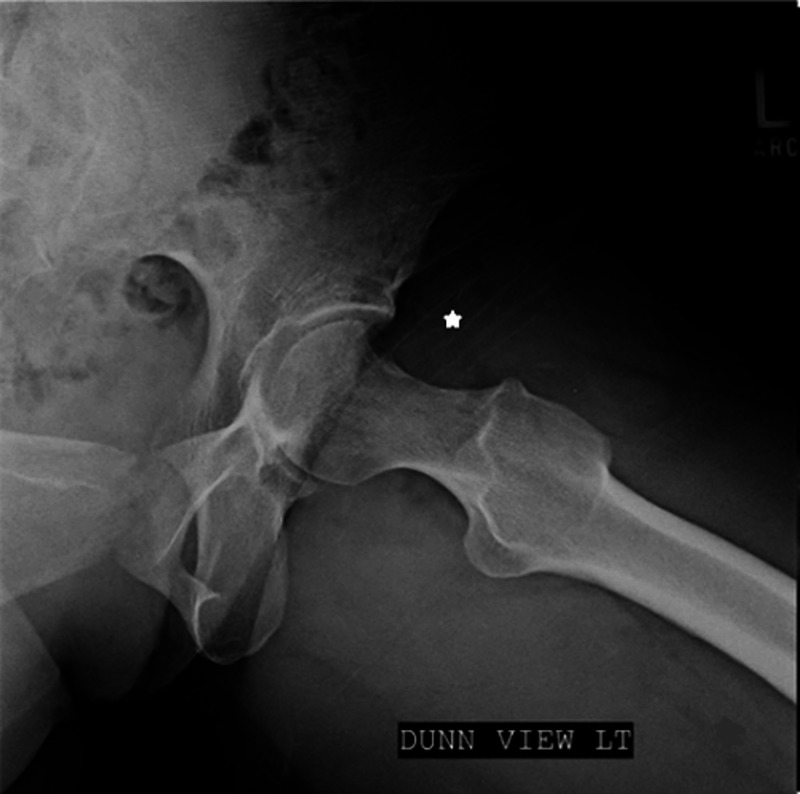
Post-Operative Radiograph Case 1 Modified Dunn radiograph taken 6 months postoperatively. The star indicates that there has been no recurrence of HO.

Hip arthroscopy was performed using the modified supine position, with general anesthetic and post-free distraction technique previously described by the senior author [12]. Arthroscopic excision of HO, measuring 12 x 14 mm, was performed and she was found to have void of the Iliofemoral (IF) ligament (Figure 2). A capsular reconstruction with the ArthroFLEX Decellularized Dermal Allograft (Arthrex) was performed using modifications of previously published techniques (Figure 3a) [13]. A revision acetabuloplasty, allograft labral reconstruction, and revision femoral neck osteoplasty were also performed as indicated (Figure 3b). Intraoperative findings are shown in Table 2. Postoperatively, she was placed in a hip abduction brace which was unlocked from 0-90 degrees of flexion when ambulating and locked in extension while sleeping for 4 weeks. Rehabilitation included 2 weeks of 20-pound flat foot weightbearing and therapy progressed using our standard labral repair protocol. She was also treated with 500 mg Naproxen twice a day for 2 weeks for HO prophylaxis. Post-operative radiographs at 6 months showed no recurrence of the HO (Figure 4). Patient reported outcome measures (PROMs) at 6 months postoperatively showed that she had a significant decrease in DP, PI, and PIY. She also had a significant increase in PF and SR. There was no significant difference in the preoperative and postoperative scores for AX and GH (Table 3). At the 1 year follow up, she reported no recurrence of hip pain. 

**Table 2:** Intraoperative Findings

Intraoperative Findings	Patient 1	Patient 2
Acetabulum (Beck Grade)		
Zone 2	1	1
Zone 3	1	1
Zone 4	1	0
Femoral Head (Outerbridge Grade)		
Zone 4	2	0
Labral tear length A-P clock face	2 - 10 o'clock	4 - 8 o'clock
Anchors used	8	7
Labral Recon Graft Type	IT band allograft	IT band allograft
Capsular Recon Graft Type	Dermal allograft	Dermal allograft
Procedures performed		
Femoroplasty	1	1
Labral repair	1	1
Labral Reconstruction	1	1
Capsular Reconstruction	1	1

Case 2

**Figure 5:**
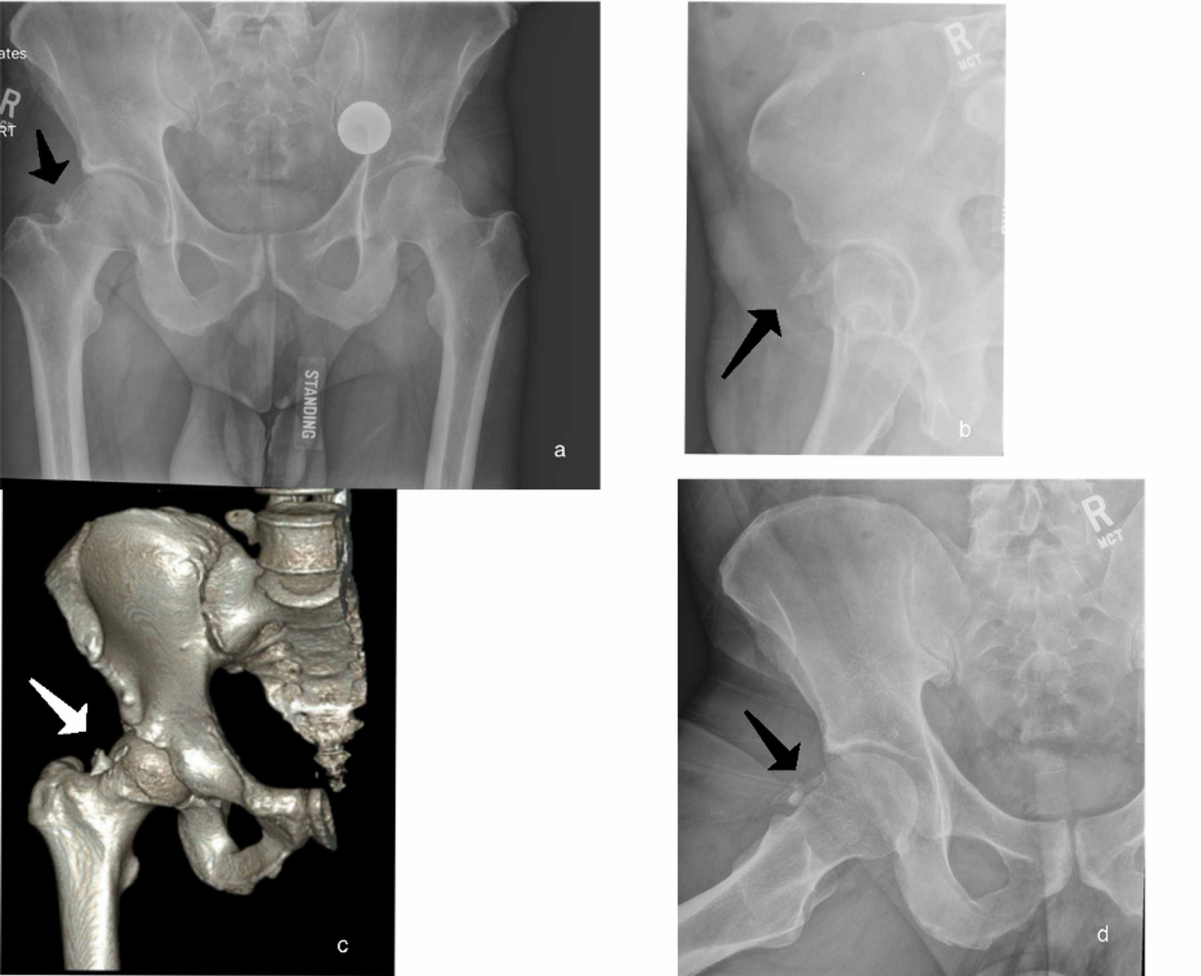
Case 2: Preoperative Imaging (a) AP pelvis with HO seen on the left hip (arrow). (b) Modified Dunn view showing grade 1 HO in the hip capsule (star). (c) False-profile view showing HO (arrow). (d) 3D reconstruction CT-image of the left hip demonstrating HO (star).

**Figure 6:**
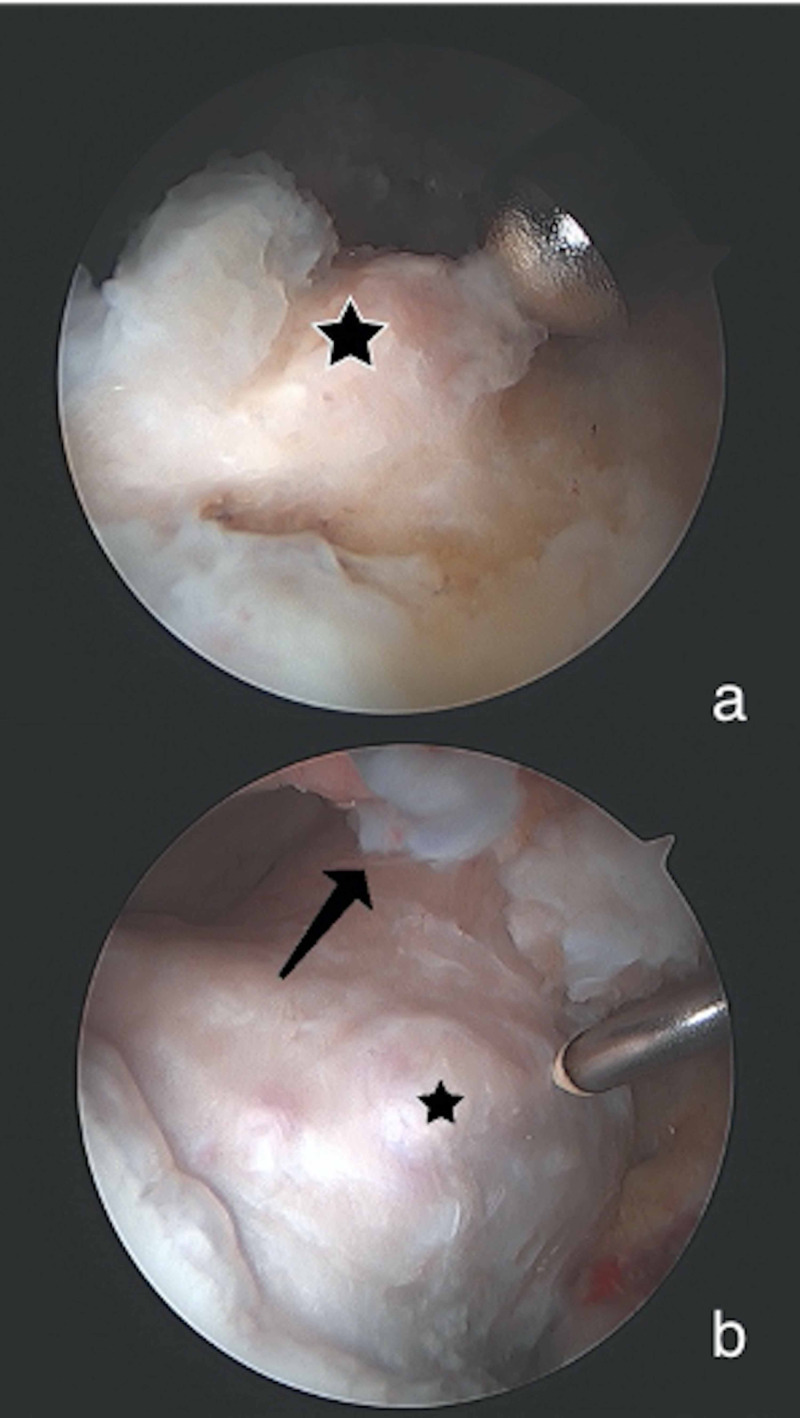
Arthroscopic Findings From Case 2 All images are of a right hip and are viewed from an anterolateral portal. (a) Heterotopic bone (star) which is intrasubstance within the capsule. (b) Remnant iliofemoral ligament, distally (arrow) after HO. The star indicates residual structural disease on the femoral neck.

**Figure 7:**
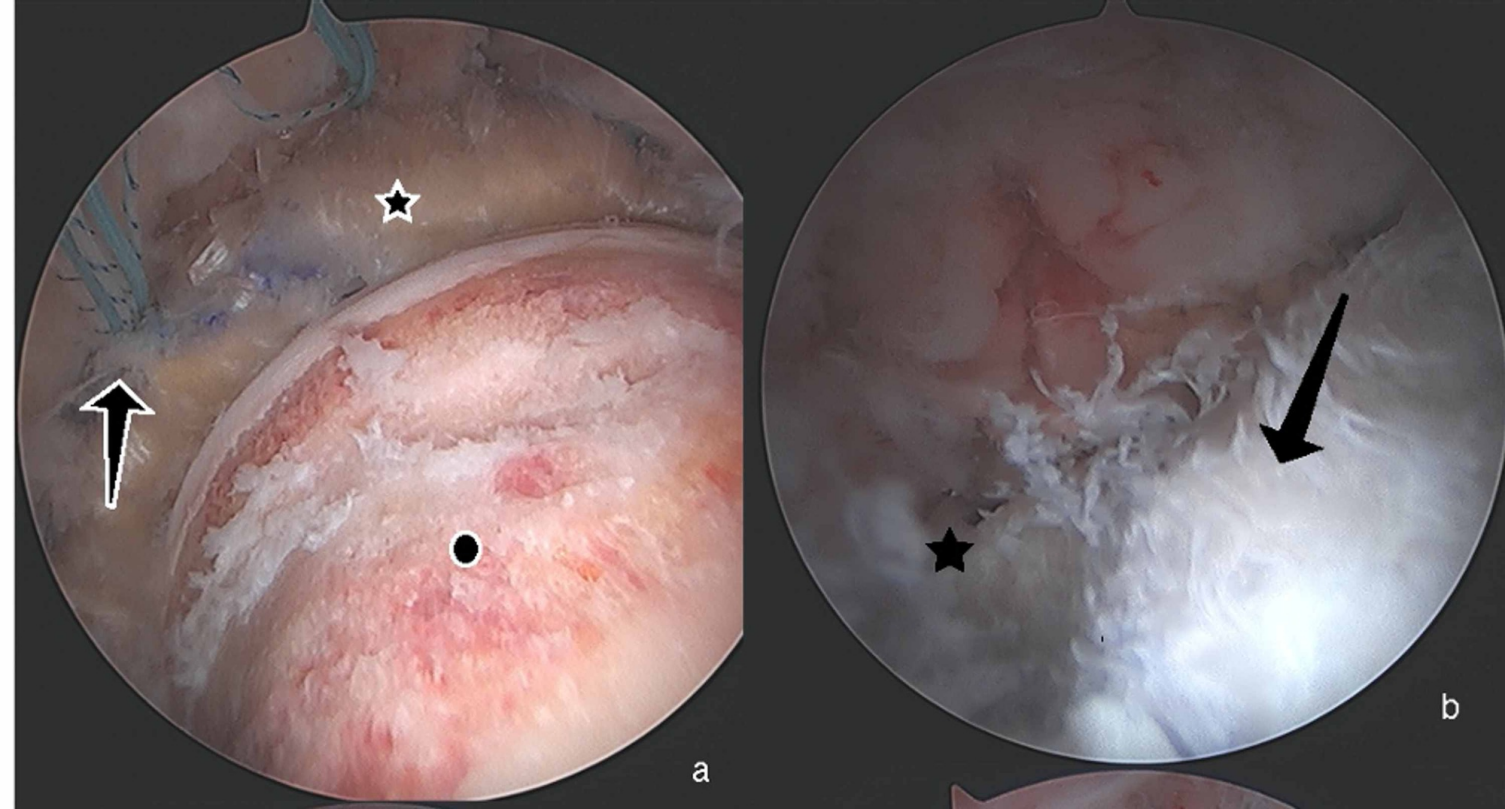
Arthroscopic Images From Case 2 Images are of a right hip (a) Completed labral allograft reconstruction (star), viewed from the midanterolateral portal, with acetabular anchors for capsular reconstruction (arrow) and revision femoral neck osteochondroplasty (circle). (b) Viewing from the anterolateral portal, the star designates the proximal acetabular side of the dermal graft; the arrow indicates the dermal allograft reconstruction.

**Figure 8:**
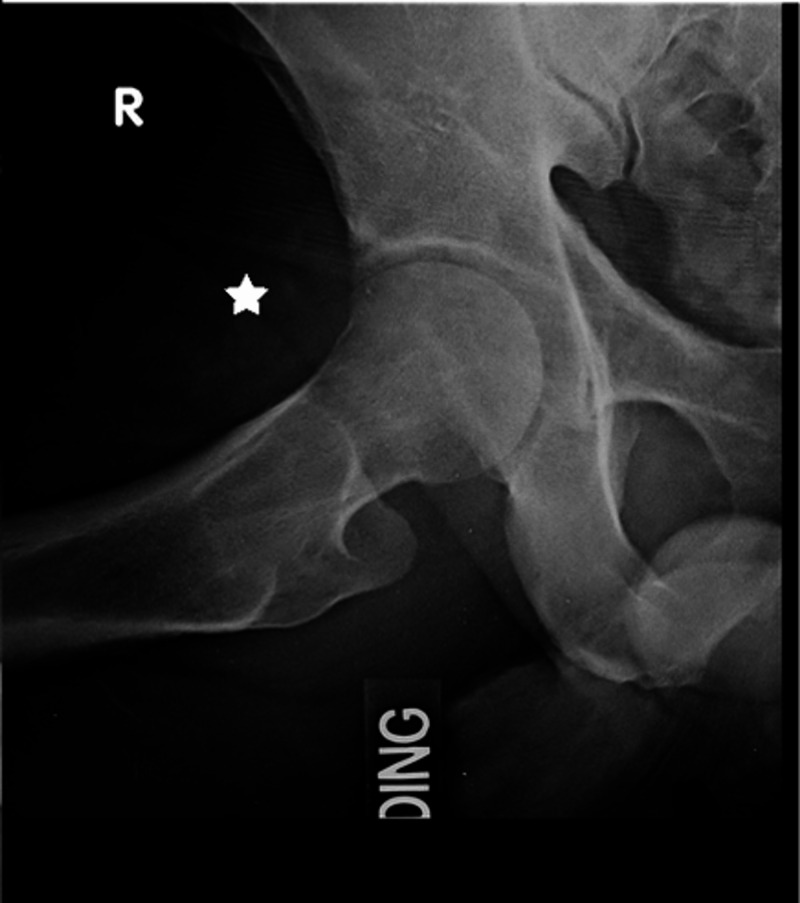
Post-Operative Radiograph Case 2 Modified Dunn radiograph taken 6 months postoperatively. The star indicates that there has been no recurrence of HO.

A 43-year-old male, with a history of two prior hip arthroscopies by an outside surgeon, presented with persistent right hip pain. He had previously tried therapy without resolution of pain. His exam showed a synovitic hip, decreased ROM, positive FADIR, and evidence of instability with a decreased spring back sign and increased external rotation at 90 degrees of hip flexion. Preoperative imaging to include plain radiographs and a 3D CT showed residual structural disease and HO which was Brooker Grade 1 (Figure 5). Radiographic findings are shown in Table 1. After discussion of treatment options, the patient elected to proceed with revision hip arthroscopy. PROMIS CAT measures were obtained preoperatively as described in Case 1. Hip arthroscopy was performed using the techniques above [12]. Arthroscopic excision of HO, measuring 3 x 4 cm, was performed and he was found to have void of the IF ligament (Figure 6). Capsular reconstruction was performed as described above. A revision acetabuloplasty, allograft labral reconstruction, and revision femoral neck osteoplasty were also performed as indicated (Figure 7b). Intraoperative findings are shown in Table 2. The patient followed the postoperative course described above. Post-operative radiographs at 6 months showed no recurrence of the HO (Figure 8). At 6 months postop, the PROMIS CAT measures of GH and PIY showed no significant change. His postoperative scores for PF, AX, DP, PI, and SR were not obtained due to survey error at the 6-month interval (Table 3). At the 1 year follow up, he reported improvement in his hip pain when compared to his preoperative state.

**Table 3:** PROMs from PROMIS Scores Global Health (GH); Significant PROMIS changes, greater than 1 Standard Deviation, indicated by asterisks (*)

	Patient 1			Patient 2		
Outcome Measure	Pre-Op	6 Months Post Op	Difference	Pre-Op	6 Months Post Op	Difference
Anxiety	52.36	43.57	-8.79	54.37		
Depression	49.91	34.17	-15.74^*^	54.32		
Pain Interference	60.3	48.73	-11.57^*^	66.87		
Social Roles	49.59	67.52	+17.93^*^	49.59		
Physical Function	40.14	53.57	+13.43^*^	38.9		
GH Mental	59.9	53.3	-6.6	38.8	45	+6.2
GH Physical	50.8	57.7	+6.9	39.8	42	+2.2
Pain Intensity	52	30.7	-21.3^*^	54.5	57	+2.5
Satisfaction		10				

## Discussion

While intracapsular HO has previously been reported, the patient had a high Harris hip score and no treatment was indicated [2, 14]. We present the only reported cases with radiographic evidence of symptomatic intracapsular HO formation following hip arthroscopy requiring intervention. We believe this radiographic finding may serve as an indicator of capsular deficiency after excision with revision hip arthroscopy. 

HO has been reported after acetabular trauma, total hip arthroplasty (THA), and hip arthroscopy [15]. Risk factors for HO after hip arthroscopy vary by study but generally consist of osteoplasty of acetabulum and femoral neck along with male sex [16, 17]. HO after hip arthroscopy most commonly forms at the anterior and lateral aspects of the hip. Anterior HO tends to be more symptomatic as it more frequently results of impingement during hip flexion leading to pain. Anterior HO tends to be more often associated with capsular involvement and lateral HO within the abductor musculature [1]. Both of our patients had anterior based HO which was found to be intracapsular upon revision arthroscopy. This was appreciated on preoperative modified Dunn and false profile radiographs as well as MRI and CT scans. We found the CT scan with 3D reconstructions to be of value when assessing prior femoral neck osteoplasties and localizing HO for preoperative planning. 

There is currently inconclusive data on whether capsular repair influences rates of HO. In 2015, Amar et al. compared capsular repair and non-repair in 100 patients in a retrospective study and found no difference HO rate [18]. However, the Beckmann et al. study in 2014 showed some trend towards HO in patients without capsular repair, however no statistical significance was achieved [16]. In their 2013 study, Campbell et al. described a 27-year old male who underwent open excision of symptomatic anterior and anterolateral HO, secondary to hip arthroscopy. Three months after removal of the HO the patient was found to have symptomatic capsular deficiency. Ultrasound examination revealed a three cm defect in the capsule which was treated with three platelet rich plasma injections and two bone marrow aspirate concentrate injections over a period of 16 weeks. This finding adds to the importance of proper capsular management and closure during hip arthroscopy [8]. A systematic review by Westermann et al. in 2018 also failed to find conclusive evidence for capsular closure and HO rates in primary hip arthroscopy; they did however recommend consideration of capsular repair in revision cases, extensile capsular releases, and in athletes returning to sports [19]. The practice at our institution is capsular repair, and two weeks of Naproxen (500mg BID) as part of both primary and revision hip arthroscopies.

## Conclusions

Our goal was to describe the radiographic findings and treatments with regard to HO. Given the limited reports on outcomes on excision of symptomatic HO as well as capsular reconstruction, we feel this finding will be relevant to hip preservationists. This study has several limitations. First is that it includes only two patients with this radiographic finding. However, both required capsular reconstruction upon removal of the heterotopic bone. Second is the short-term follow-up of outcome measures. Despite these limitations, we believe this radiographic finding may be useful in the preoperative diagnosis of capsular deficiency suggesting that a capsular reconstruction may be indicated during revision hip arthroscopy. When presented with this finding, hip arthroscopists should consider capsular reconstruction in the revision setting.
